# Glycyrrhizic Acid Relieves Fatigue Induced by Acute and Chronic Exercise in Mice via Inhibition of the NF‐κB/NLRP3 Signaling Pathway

**DOI:** 10.1002/brb3.71773

**Published:** 2026-09-27

**Authors:** Xin‐ying Cai, Wen‐jun Li, Cai‐xia Chen, Lu Peng, Li‐chao Yang, Xiao‐ning Lin, Hong‐tao Liu, Jian‐bo Jia, Yan‐chao Jiao, Ying Li

**Affiliations:** ^1^ School of Medicine Xiamen University Xiamen People's Republic of China; ^2^ Department of Neurosurgery, Zhongshan Hospital of Xiamen University, School of Medicine Xiamen University Xiamen People's Republic of China; ^3^ Department of Pharmacy The First Hospital of Hebei Medical University Shijiazhuang People's Republic of China; ^4^ Department of Breast Surgery the Affiliated Zhongshan Hospital of Xiamen University,School of Medicine, Xiamen University Xiamen People's Republic of China; ^5^ Department of Otolaryngology the Affiliated Zhongshan Hospital of Xiamen University, School of Medicine, Xiamen University Xiamen People's Republic of China; ^6^ Bokang Bioengineering (Shandong) Co., Ltd Linyi People's Republic of China; ^7^ Shandong Hankang Medical Technology Group Co., Ltd Linyi People's Republic of China; ^8^ Department of Pharmacy Xiamen Medical College Xiamen People's Republic of China

**Keywords:** apoptosis, chronic fatigue syndrome, fatigue, glycyrrhizic acid, NF‐κB, NLRP3

## Abstract

**Background:**

Glycyrrhizic acid (Gly‐A) exhibits anti‐inflammatory and antioxidant effects in various central nervous system disorders. Acute fatigue and chronic fatigue (CF) are associated with serious physical and psychological effects. However, the underlying pathophysiology and effective therapeutic strategies for these conditions remain poorly understood.

**Purpose:**

This study aims to evaluate the therapeutic potential of Gly‐A against fatigue, and to elucidate the underlying mechanisms of its action.

**Methods:**

We established a mouse model of acute exercise‐induced fatigue and evaluated fatigue‐related indices. To identify specific pathways involved in Gly‐A's therapeutic effects, we employed RNA sequencing, network pharmacology, and in vitro oxygen‐deficit models, aiming to clarify the mechanisms. Additionally, we established a mouse model of CF to compare the therapeutic efficacy of Gly‐A with positive control drugs by assessing identical parameters.

**Results:**

Gly‐A significantly improved fatigue‐related indices without affecting body weight. RNA sequencing and network pharmacology analyses showed that Gly‐A modulates oxidative stress responses and targets the NF‐κB (nuclear factor‐kappa B)/NLRP3 (NOD‐like receptor thermal protein domain associated protein 3) signaling and apoptotic pathways. In the CF mouse model, oral administration of Gly‐A significantly increased motor activity, alleviated anxiety and depression, and improved neurocognitive function compared to model mice, exhibiting superior efficacy to positive control drugs. These findings suggest that Gly‐A exerts anti‐inflammatory effects by targeting the NF‐κB/NLRP3 signaling pathway, thereby reducing neuronal apoptosis in CF.

**Conclusions:**

Gly‐A exhibits therapeutic potential against acute exercise‐fatigue and CF, primarily by attenuating apoptosis through the inhibition of the NF‐κB/NLRP3 signaling pathway.

## Graphical abstract

Molecular mechanism diagram of Gly‐A in the treatment of acute and chronic exercise‐fatigue.

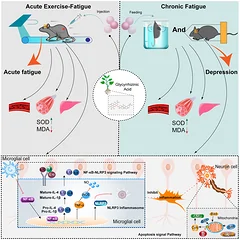

## 1 Introduction

Fatigue, characterized by impairments in motor performance and diminished mental alertness, results from energy depletion (Lee et al. 2022), metabolite accumulation (Wiehler et al. 2022), and oxidative stress (O'Connor et al. 2022). Its persistence and unpredictability impose substantial burdens on both patients and society (Clark et al. 2016). Acute fatigue, when compounded by inadequate recovery, progresses to chronic fatigue (CF), which is a condition characterized by impaired cognitive and physical performance, as well as adverse effects on physiological health and subjective well‐being (Jane et al. 2020). When symptoms persist untreated for extended periods, individuals may develop chronic fatigue syndrome (CFS), transitioning from a healthy state to one of chronic suboptimal health (Grach et al. 2023).

The NOD‐like receptor pyrin domain containing 3 (NLRP3) inflammasome has received increasing attention in the context of fatigue. As a key component of the innate immune response, the NLRP3 inflammasome regulates the release of interleukin‑1β (IL‑1β) upon cellular injury (Kelley et al. 2019). Its activation is driven by the nuclear factor kappa B (NF‑κB) signaling pathway (S. Li et al. 2022). Mitochondrial dysfunction and ROS are regarded as hallmarks of damage of skeletal muscle in exercise‐fatigue (H. Zhang, Zhao, et al. 2022), and heightened NLRP3 activity coupled with impaired immune defenses correlates with CF (Z. T. Zhang et al. 2016). In the central nervous system, microglia serve as the primary innate immune cells, mounting rapid defenses against harmful stimuli. Upon activation, microglia polarize into pro‑inflammatory (M1) or anti‑inflammatory (M2) phenotypes (Borst et al. 2021). Emerging evidence suggests that NLRP3‑driven M1 polarization during strenuous exercise contributes to chronic inflammation, which may underlie CFS pathogenesis (X. Wang et al. 2018). Overall, the NF‑κB/NLRP3 axis represents a critical immune defense mechanism implicated in both acute fatigue and the development of CF.

To date, the pathophysiology of acute and chronic exercise‑fatigue remains poorly understood. As reported, dietary supplements such as taurine (TA) (McLeay et al. 2017), ginseng (Finsterer and Mahjoub 2014), and rhodiola (Hou et al. 2020) are commonly used to alleviate fatigue. CF‐related symptoms are often managed with pharmaceutical interventions, including testosterone, immunostimulants, corticosteroids, antidepressants, analgesics, and antihistamines (X. Wang et al. 2018). These interventions often yield unsatisfactory results due to adverse side effects and high costs (Grach et al. 2023). Consequently, there is an urgent need for more effective and safer dietary supplements or medications for acute and chronic exercise‑fatigue. Glycyrrhizic acid (Gly‑A), a triterpenoid compound derived from licorice, is renowned in traditional Chinese medicine for its anti‑inflammatory properties (Tucker et al. 2021). Recent studies have demonstrated that Gly‑A also possesses antibacterial, anticancer, immunomodulatory, and neuroprotective activities (Rehman et al. 2020; F. Zhang, Yin, et al. 2022). With regard to the pathophysiology of acute exercise‐fatigue, previous research (Hill et al. 2020) has demonstrated that following 1 h of treadmill exercise at an intensity corresponding to 65% of maximal oxygen consumption, systemic tissues may be in hypoxia state. Intense exercise can induce an acute reduction in cardiac function (Stewart et al. 2017). This transient decline in cardiac function may contribute to the hypoxic state observed during high‐intensity physical exertion (Kleinnibbelink et al. 2021). Reviewing previous research indicates that Gly‐A has demonstrated efficacy in neurological disorders such as stroke, Alzheimer's disease, and epilepsy (Chen et al. 2020; Gaikwad et al. 2021; Y. J. Li et al. 2019). However, there is relatively less research on how Gly‑A shows therapeutic effects on acute and chronic exercise‑fatigue. We hypothesize that Gly‑A exerts a beneficial therapeutic effect on both acute and chronic exercise‑fatigue. Therefore, this study aims to achieve the following objectives: (1) examine the potential effect of Gly‐A in mitigating acute and chronic exercise‑fatigue; (2) investigate the underlying mechanisms; and (3) explore the recommended route of administration in mice models to inform future clinical trials.

This study aimed to evaluate the therapeutic effect of Gly‑A for acute and chronic exercise‑fatigue in mice, and to elucidate the molecular mechanism. We employed RNA sequencing, network pharmacology, and molecular docking analyses to predict Gly‑A's molecular targets and signaling pathways, then validated its anti‑fatigue effects.

## 2 Materials and Methods

### 2.1 Chemical and Reagent

Gly‐A was provided by Chengdu Must Bio‐Technology Co. LTD. Nandrolone decanoate (Nandro) and TA were provided by Shanghai Macklin Technology Co., LTD. Formoterol fumarate (Formo) was provided by Shanghai Aladdin Biochemical Technology Co., LTD. SOD, MDA, L‐LDH, and CK assay kits were obtained from Beijing Solaibao Technology Co. LTD. The BCA protein assay kit and SDS‐PAGE gel kit were obtained from Shanghai Yisheng Biotechnology Co., LTD. Antibodies for iNOS, NLRP3, NF‐κB, Bax, Bcl‐2, Caspase 3, and GAPDH were obtained from Proteintech. Antibodies for Iba‐1 were obtained from FUJIFILM Wako Chemical Corporation. The BV2 cell and N_2_a cell lines were purchased from Suzhou Starfish Biotechnology Co. Ltd. The cells were cultured in DMEM (high glucose) medium supplemented with 10% fetal bovine serum (Table S1).

### 2.2 Animal Models and Experimental Procedures

Male C57BL/6 mice (6–8 weeks old, weighing between 18 and 30 g) were obtained from Shanghai SLAC Laboratory Animal Co. Ltd and maintained in an SPF facility at 25 ± 2°C with a relative humidity of 45 ± 1% under a 12‐h light/dark cycle. Following a 7‐day acclimatization period, the mice were randomly assigned to six groups using the random number table method based on the body weight of animals (*n* = 10 per group): a control group (intraperitoneal injection with the same volume of normal saline), a model group (administered intraperitoneal injections of normal saline at an equivalent volume), a low dose group (receiving Gly‐A at 10 mg/kg/day via intraperitoneal injection), a medium dose group (administered Gly‐A at 30 mg/kg/day intraperitoneally), a high dose group (treated with Gly‐A at 100 mg/kg/day via intraperitoneal injection), and a positive control group (Chai et al. 2022) (given TA at 500 mg/kg/day by intragastric gavage, *n* = 10).

### 2.3 Establishment of Acute Exercise‐fatigue Model

The acute exercise‐fatigue model was established by subjecting mice to forced treadmill running. The mice were placed on the treadmill for forced running, during which an electric stimulus of defined intensity was applied to ensure continuous exercise. When the treadmill records that the mouse has received the prescribed number of electric shocks within the specified time, it indicates that the animal has reached a state of physical exhaustion (electric intensity: 1.0 mA; exhaustion indicator: 15 shocks/10 s; acceleration: 5 V/s; speed: 16 m/min). On the same treadmill, mice ran until exhaustion at a fixed submaximal index over five days, with no incline (Lønbro et al. 2019). On the first day, the treadmill was set to a speed of 16 m/min, and all animals completed the full exercise session without any external handling or pacing assistance. Immediately after exercising, the designated drug treatment was administered. On the second day, the identical group performed the same exercise bout, but this time a standardized pacing protocol was implemented throughout the run. The rotarod test was performed in 30 min after drug treatment.

### 2.4 Rotarod Experiment

The rotarod test is a well‐established method for assessing motor function and coordination in mice. The animals were placed on a rotating rod that accelerated gradually from rest to a predetermined speed. The latency to fall was recorded, with a maximum trial duration of 5 min. In the rotarod experiment, the animals were preselected in a training session 24 h before the experiment to remain on the bar (at 40 rpm) for 5 min. All animals were treated with vehicle, Gly‐A (10, 30, and 100 mg/kg; i.i.), or TA (500 mg/kg; i.p.). TA, administered as a positive control agent in the treatment of acute exercise‐induced fatigue, was used to compare the therapeutic efficacy of Gly‐A. Animals were placed with all four paws onto the bar in 30 min after drug treatment, and the fall‐down latency was evaluated (Rabie et al. 2023).

### 2.5 CF Mouse Model Establishment and Agent Treatment Procedure

CF model mice (*n* = 8) were subjected once daily for three weeks to two consecutive stressors: cold‑water swimming (15 ± 1°C) for 2 h, followed immediately by 2 h of restraint stress. The model of restraint stress was established by confining mice in 50‐mL centrifuge tubes to restrict movement. Prolonged swimming induced physical exhaustion in mice. The restraint stress induced a negative emotional state characterized by heightened tension and anxiety in mice. A separate control group (*n* = 8) that received no stimuli was defined as the quiet group. Model establishment was confirmed by a swimming‑to‑exhaustion test and a forced swimming test (FST). On day 1, all mice were weighed and randomly assigned (*n*  =  8 per group) to receive by intraperitoneal injection: saline (model group), Nandro (38 mg/kg/day) (Ben‐Zeev et al. 2025), Formo (25 µg/kg/day) (Harcourt et al. 2007), or Gly‑A (100 mg/kg/day). Nandro, used as a positive control agents for the treatment of CF‐related symptoms, was administered to compare the therapeutic efficacy of Gly‐A. A fifth group received Gly‑A (100 mg/kg/day) orally. The quiet group was injected with saline for 23 days. Behavioral assessments—FST, exhaustive swimming, pole test, open‑field test, and Y‑maze—were performed on days 21–23. On day 24, following final weighing, blood was collected via the tail vein. Successful establishment of the CF model requires that the model group demonstrates marked physical exhaustion and emotional distress relative to control mice, with statistically significant differences observed in behavioral test outcomes. Mice were then euthanized, and brain, liver, and muscle tissues were rapidly harvested for further analysis (Fig 6A).

### 2.6 Forced Swimming Test

The FST is often employed to assess anxiety and depression states in mice. Swimming tests were performed in individual cylindrical tanks (60 cm × 45 cm) filled with desaturated water maintained at 30 ± 1°C; ambient temperature was held constant at 22 ± 1°C. Following body weight measurement, a calibrated metal load was affixed to the base of each mouse's tail using elastic, non‐traumatic tape. Exhaustion was defined as failure to maintain surface buoyancy, loss of coordinated limb movement during swimming, or continuous submersion exceeding 10 s (Potolitsyna et al. 2023). Mice demonstrating depression‐like behavior display prolonged immobility or significantly reduced locomotor activity during forced swim test exposure. The experimental mice were exposed to forced swimming (except the quiet group) to induce chronic physical fatigue and an anxious spirit. Animals were forced to swim for 2 h every day for 21 days at room temperature. After initial vigorous activity, each animal assumed a typical immobile posture intermittently with complete cessation of movements. On the 21st day, after swimming for 2 min, the total duration of immobility, low action, and high action in seconds in the next 5 min was observed. After recording the immobility duration of forced swimming, our group recorded the total duration of exhausted swimming of experimental mice. Drugs were administered 1 h before the test on each day.

### 2.7 Pole Test

The pole test is employed to assess motor coordination in mice. The pole test apparatus consisted of a 50 cm‑high vertical pole (1 cm diameter) topped with a 2.5‐cm‑diameter sphere. The pole was positioned at a 90° angle. Each mouse was placed upright on the sphere and allowed to descend under its own volition. Two timings were recorded: the time to descend from the top to the base (T‑Time) and the latency to complete a 180° turn (T‑Turn). Each mouse underwent three independent trials, and the average of those trials was used for analysis.

### 2.8 Open‐Field Test

The open‐field test (OFT) serves as a standardized behavioral paradigm to quantify spontaneous locomotor activity and detect anxiety‐ or depression‐like behaviors in mice introduced to a novel environment. Mice displaying anxiety‐related phenotypes consistently demonstrate diminished exploratory activity and reduced mobility in the center zone. OFT behavioral assessment was conducted in an open‑field arena (100 cm × 100 cm), with the central zone occupying 36% (9/25) of the total area. Each mouse was placed in the area and allowed to explore for 5 min, during which the number of central‑zone entries and crossings was recorded. All open‑field test parameters were automatically captured and analyzed by the Smart Recording System.

### 2.9 Y‐Maze Behavioral Test

The Y‐maze test utilizes rodents' innate exploratory behavior to assess cognitive function, with spontaneous alternation performance serving as a reliable indicator of intact short‐term spatial working memory. Reduced alternation triplet and decreased number of arm entries directly reflect working memory deficits in this paradigm. Each mouse was placed at the end of the S arm, facing away from the center, and allowed to explore the Y‑maze for 6 min. Behavior was recorded and tracked by the Smart Recording System. An arm entry was scored each time all four paws completely entered an arm. Trials began immediately upon placement and ended automatically at 6 min, with all arm entries captured and analyzed by the system.

### 2.10 Hematoxylin–Eosin Stain

After tissue collection, muscle and liver tissues were rinsed three times before being processed into paraffin‐embedded sections. These sections were then stained using an HE Stain Kit and subsequently examined under a microscope (R. Sun et al. 2025). Hematoxylin–Eosin (HE) staining was used to evaluate the histomorphology of liver and muscle tissues. Muscle morphology serves as a physiological index for assessing muscle function, while liver toxicity is a key indicator for evaluating drug‐induced adverse effects.

### 2.11 Immunofluorescence Analysis

Mice were anesthetized with chloral hydrate and transcardially perfused with ice‐cold saline, then fixed with 4% paraformaldehyde. Brains were removed, dehydrated in graded sucrose solutions (10%, 20%, 30%), and sectioned coronally at 30 µm using a vibrating microtome. Sections were blocked in PBS with 0.5% Triton X‐100 and 10% normal goat serum for 1 h at room temperature, then incubated overnight at 4°C with primary antibodies against NF‐κB or Iba‐1. After PBS rinses, sections were incubated with goat anti‐rabbit or goat anti‐mouse secondary antibodies. NF‐κB and Iba‐1 immunofluorescence (IF) intensities were analyzed by fluorescence confocal microscopy and quantified in three randomly selected 1024 µm × 1024 µm fields in the hippocampal CA1 region. Antibodies derived from the same source were employed with a three‐standard four‐color multiplex fluorescence staining kit.

### 2.12 Network Pharmacology

A range of databases was utilized to identify and analyze potential targets associated with Gly‐A and its anti‐fatigue properties. Initially, the Traditional Chinese Medicine Systems Pharmacology Database and Analysis Platform (TCMSP) was employed to predict potential targets for bioavailable Gly‐A based on its chemical structure. These predicted targets were subsequently validated using information from the Online Mendelian Inheritance in Man (OMIM), GeneCards, and DisGeNET databases. Moreover, interactions between fatigue‐associated targets and Gly‐A's active compounds were mapped using the STRING database. Finally, Gene Ontology (GO) and Kyoto Encyclopedia of Genes and Genomes (KEGG) enrichment analyses were performed and visualized for fatigue‐related targets with the assistance of the DAVID database.

### 2.13 Molecular Docking

The affinity of Gly‐A toward the STAT3 pyrin domain (PYD) (PDB ID: 6NJS), DRD2 PYD (PDB ID: 6CM4), MMP9 (PDB ID: 1ITV), and BCL2‐L (PDB ID: 5UUK) was analyzed using Autodock Vina and PyMol. Autodock Vina and PyMol 2.6.0 were used to examine and exhibit the process of compound‐target molecular docking and binding affinity, with a critical affinity value of −5.0 kcal/mol (Wen et al. 2025).

### 2.14 Drug Toxicity Test and Oxygen Deficit Cell Culture

During high‐intensity exercise, the body often lacks sufficient oxygen, leading to hypoxia that causes shortness of breath, dizziness, and muscle soreness (Hill et al. 2020). In this study, we applied the oxygen deficit (OD) model to develop an in vitro neuronal cell model that replicates a fatigued state. BV2 and N2a cells were seeded after 12 h, and the original medium was replaced with fresh culture medium supplemented with a predetermined drug concentration. For the OD treatment, cells were exposed to various drug concentrations and maintained for several hours. The incubation was carried out at 37°C in a controlled atmosphere consisting of 94% N_2_, 5% CO_2_, and 1% O_2_. At designated time intervals, cell viability was measured using the CCK‐8 assay.

### 2.15 Western Blot (WB) Analysis

Mouse brain tissues were lysed in lysis buffer, centrifuged at 16,000 *g* for 15 min, and the supernatant was collected and boiled in SDS loading buffer for analysis. Proteins were separated by SDS‐PAGE, transferred to a membrane, and detected using specific primary and secondary antibodies.

### 2.16 RNA Sequencing

RNA sequencing was conducted using complete hippocampal tissue from Gly‐A treatment group (100 mg/kg) and model group. The trizol reagent was used to extract total RNA. RNA sequencing was sequenced using Illumina PE150. Data were presented as reads per kilobase million, and datasets were analyzed to identify upregulated and downregulated pathways.

### 2.17 qRT‐PCR

Tissues or cells were collected, and total RNA was extracted using Trizol reagent. RNA concentration was quantified via spectrophotometry. Real‐time quantitative PCR was performed in a 20 µL reaction system containing SYBR Green I dye, with gene expression levels normalized to GAPDH as the internal reference. Amplification was carried out in 96‐well plates on a Bio‐Rad Real‐Time PCR System according to the manufacturer's instructions. Gene expression levels were analyzed using the ΔΔ*Ct* method (Table S2).

## 3 Statistical Analysis

Statistical analysis was performed using GraphPad Prism, and results are expressed as the mean ± SEM. Group differences were evaluated by one‐way ANOVA, except for the rotarod test data, which were analyzed with repeated measures ANOVA. An independent‐samples *t*‐test was conducted to compare the means of the two groups. One‐way ANOVA is a statistical procedure used to evaluate the effect of a single independent variable (factor) on a dependent variable across three or more distinct levels (groups), while repeated measures ANOVA is used to analyze data obtained from multiple measurements collected on the same subjects across different time points or experimental conditions. In our study, the rotarod data were collected and analyzed on a daily basis throughout the experimental period, whereas the data from other behavioral tests and molecular biology assays reflect the relationship between a single independent variable and the corresponding dependent variable. Tukey's post hoc test was employed for pairwise comparisons, and a *p*‐value below 0.05 was deemed statistically significant.

## 4 Results

### 4.1 Gly‐A Significantly Inhibited Acute Exercise‐Fatigue in the Mice Model

In pilot experiments, intraperitoneal injection at 100 mg/kg significantly improved physical performance relative to the vehicle control in an acute exercise‐fatigue model, whereas intragastric administration yielded no statistically significant effect in the rotarod test. We first determined the administration route of Gly‐A as intraperitoneal injection, with a dosage of 100 mg/kg (Figure 1A). Then, the experiment was conducted following the protocol depicted in Figure 1B; an acute exercise‐fatigue model was established in mice by subjecting them to forced running. The successful induction of acute exercise‐fatigue was validated by a marked decrease in the duration that mice remained on the rotarod compared to the control group (CTL). After Gly‐A treatment (Figure 1C), there was a significant recovery in fatigue indicators, including time and distance to exhaustion, which increased significantly in the Gly‐A (100 mg/kg) group during the forced running experiment (Figure 1D,E). Additionally, both the Gly‐A (30 and 100 mg/kg) and TA groups showed increased staying time on the bar in the rotarod experiment (Figure 1F). In contrast, mice receiving TA treatment experienced a pronounced reduction in body mass, while Gly‐A administration did not result in any significant change in weight (Figure 1G). Therefore, these results showed that Gly‐A exhibits significant anti‐fatigue effects in mice.

**FIGURE 1:**
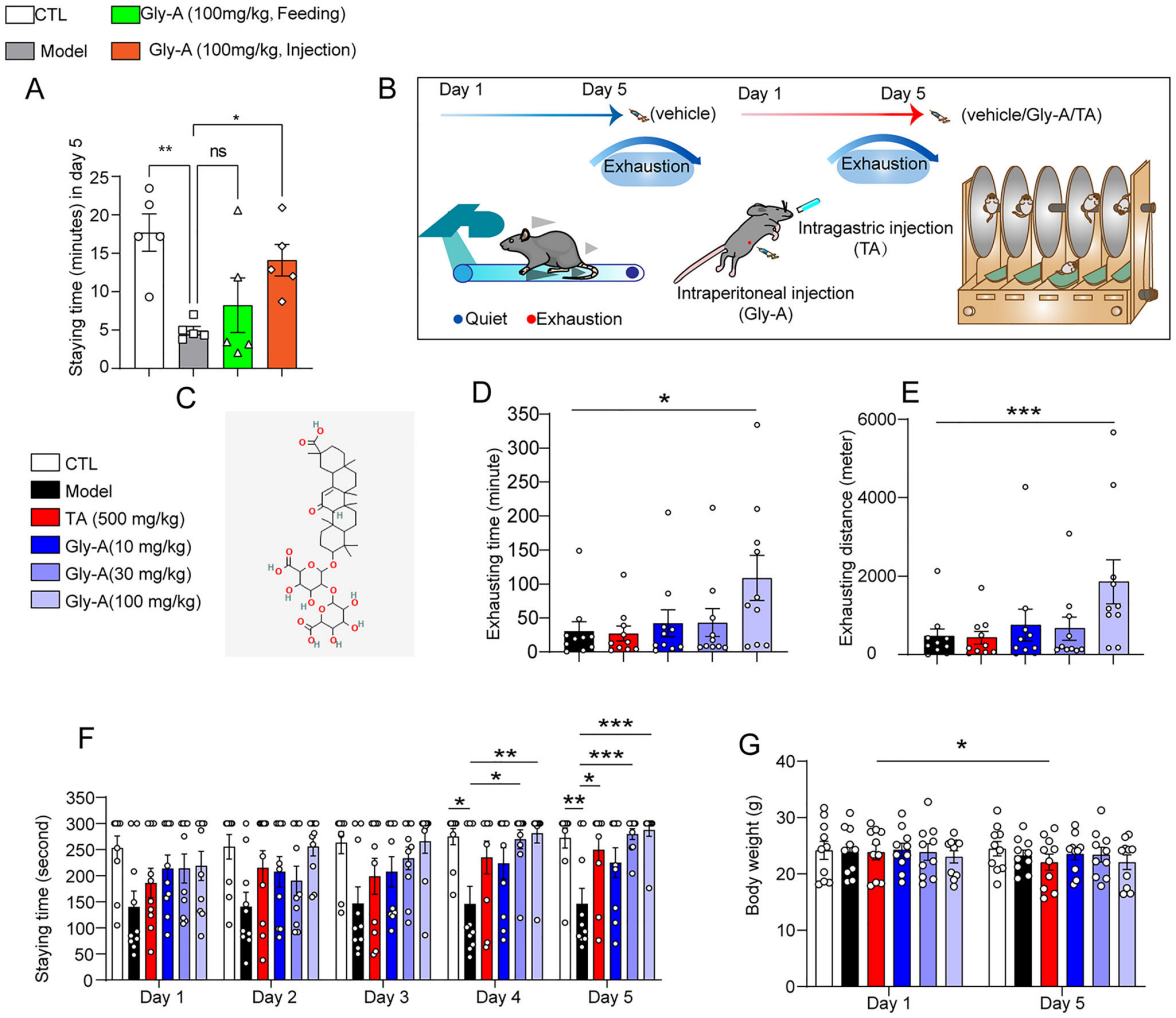
Gly‐A significantly inhibited fatigue in the mice model. (A) Changes of staying time in rotarod experiment in pilot experiment. (B) Experimental diagram of fatigue model construction and Gly‐A/TA administration treatment. (C) Molecular formula of Gly‐A. (D) Exhausting time at the end of fatigue model construction and Gly‐A/TA administration treatment. (E) Exhausting distance at the end of fatigue model construction and Gly‐A/TA administration treatment. (F) Changes of staying time in rotarod experiment during fatigue model construction. (G) Changes in body weight of mice. CTL, control group; Gly‐A, glycyrrhizic acid; TA, taurine (*p < 0.05, **p < 0.01, ***p < 0.001).

### 4.2 Molecular Mechanism of Gly‐A Inhibited Acute Exercise‐Fatigue

Using network pharmacology technique, we identified 29 key target genes of Gly‐A from databases. In addition, we identified 1474 related targets for fatigue. Based on the network pharmacology analysis, a Venn diagram was generated with disease‐related targets, revealing 16 key targets (Figure 2A). Subsequently, the 16 identified targets were analyzed and visualized using Cytoscape, thereby predicting their potential interconnections (Figure 2B). Cytoscape was utilized to construct a regulatory network illustrating the relationship between Gly‐A and fatigue. Furthermore, GO enrichment analysis demonstrated that these targets are implicated in xenobiotic response, the enhancement of cell proliferation, the suppression of apoptosis, and the modulation of cocaine‐related behaviors (Figure 2C). Furthermore, KEGG pathway analysis revealed notable enrichment in several signaling routes, including those related to gap junction pathways (Figure 2D). These findings suggest that these signaling pathways may underlie the anti‐fatigue effects of Gly‐A. In order to elucidate the connection between Gly‐A and its central targets, molecular docking was employed as a bioinformatics tool for predictive analysis. Molecular docking analysis quantified the interaction energies between Gly‐A's active components and five key targets—STAT3, DRD2, MMP9, Jun, and Bcl2‐L. The binding affinity was evaluated using a threshold of −5.0 kcal/mol (Figure 2E–H), a standard benchmark for assessing the stability of receptor–ligand complexes (Figure 2I).

**FIGURE 2:**
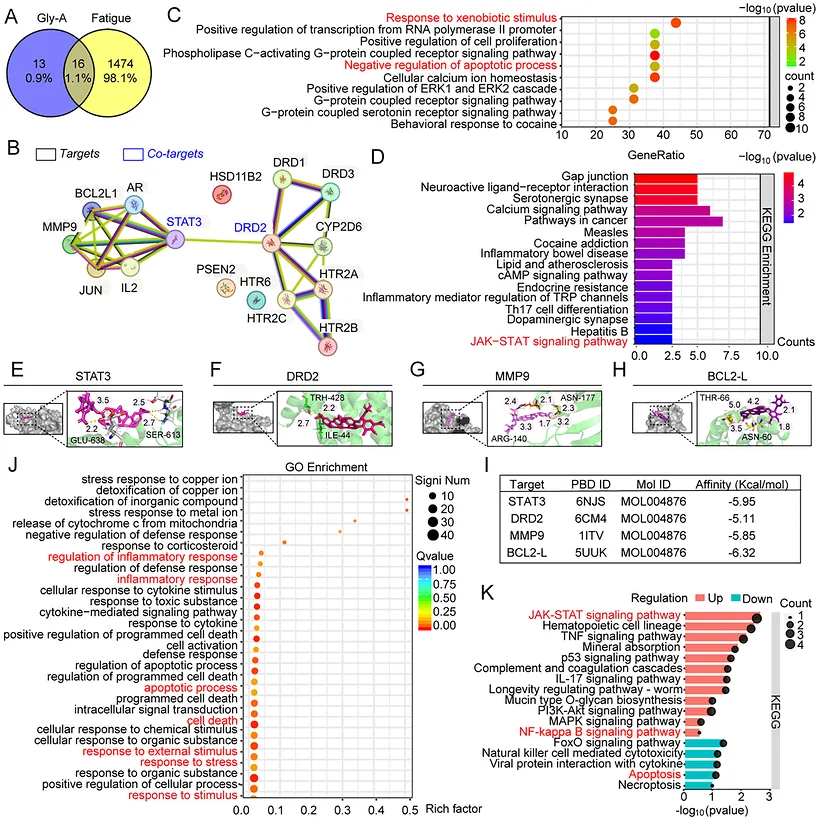
Molecular mechanism of Gly‐A‐inhibited fatigue. (A) Venn diagram of potential targets of Gly‐A against fatigue. (B) PPI network and target protein interaction network of the 16 co‐targets of Gly‐A in the treatment of fatigue. (C) GO pathway enrichment analysis of network pharmacology. (D) KEGG pathway enrichment analysis of network pharmacology. (E–H) Schematic diagram of molecular docking of Gly‐A with STAT3, DRD2, MMP9, and Bcl2‐L. (I) Molecular docking results of Gly‐A and major active ingredients. (J) GO pathway enrichment analysis of RNA sequencing in the hippocampal regions between model group and Gly‐A (100 mg/kg) group. (K) KEGG pathway enrichment analysis of RNA sequencing in the hippocampal regions between model group and Gly‐A (100 mg/kg) group. Gly‐A, glycyrrhizic acid.

Evidence demonstrates that physical and mental fatigue selectively impair critical cognitive processes with direct consequences for behavioral accuracy and safety‐critical task performance (Yao et al. 2025). To further assess Gly‐A–induced transcriptional alterations associated with fatigue‐related neurocognitive impairment, we performed RNA sequencing on hippocampal tissue from untreated and Gly‐A–treated (100 mg/kg) mice. This quantitative deep sequencing approach enabled us to measure and compare mRNA expression profiles. GO enrichment analysis showed that Gly‐A treatment for fatigue was associated with response to stimulus, response to stress, apoptotic processes, and regulation of inflammation (Figure 2J). KEGG enrichment analysis revealed that the apoptotic signaling pathway and inflammatory response pathways, including the NF‐κB and JAK‐STAT signaling pathways, were closely related (Figure 2K). These results indicate that the identified signaling pathways could be integral to Gly‐A's anti‐fatigue properties. Given the strong correlation between inflammatory response and apoptotic signaling in fatigue, we carried out additional validation studies on these pathways.

### 4.3 Gly‐A Inhibited Oxidative Stress and Enhanced Skeletal Muscle Strength In Vivo

The hepatic lobule structure was well‐preserved in the CTL, Gly‐A (10 mg/kg), and Gly‐A (30 mg/kg) groups, with clearly defined architecture and no structural abnormalities. Hepatic sinusoids exhibited a resting morphology (red), hepatocytes displayed normal morphology and uniform size (black), and no inflammatory cell infiltration was observed, indicating the absence of active inflammation. Collectively, these findings confirm the absence of significant pathological changes in these groups. In contrast, in the Model, TA, and Gly‐A (100 mg/kg) groups, the overall lobular architecture remained largely intact, and mild but consistent histopathological alterations were evident. Hepatocyte cords showed slight disorganization. Occasional cytoplasmic edema (black), scattered fat vacuoles in the parenchyma (yellow), and minimal lymphocytic infiltration within sinusoids (green) collectively indicate subtle hepatocellular injury and a low‐grade inflammatory response (Figure 3A). In the CTL group, muscle fibers (black) were uniformly sized, regularly arranged, and displayed clear striations, with no evidence of inflammatory infiltration, edema, or fibrosis, indicating normal histological architecture. In contrast, the model group exhibited extensive myofiber swelling and degeneration (black), atrophy, rupture (red), interstitial edema, and diffuse inflammatory cell infiltration (blue), consistent with severe muscular injury. Compared to the model group, the Gly‐A (10 mg/kg) group showed increased areas of fiber degeneration and scattered necrosis (black), minor structural disruption, and persistent but focal inflammatory infiltration; however, isolated regenerative changes were observed (green), suggesting limited repair activity. In the Gly‐A (30 mg/kg) group, myofibers displayed heterogeneous size and disorganized alignment (black), accompanied by mild interstitial inflammation (blue) and partial regeneration (green), indicating ongoing but incomplete recovery. The Gly‐A (100 mg/kg) group demonstrated well‐preserved muscle bundle architecture, predominantly orderly fiber arrangement, occasional degenerated fibers (black), and minimal inflammatory infiltration (blue), reflecting a marked attenuation of pathological changes. In the TA group, most muscle fibers appeared structurally intact and well‐aligned (black), with significantly reduced degeneration and necrosis, and the presence of regenerating fibers characterized by basophilic cytoplasm and large, centrally located nuclei (green), together with substantially diminished inflammatory infiltration (blue), collectively indicating effective tissue protection and active regeneration (Figure 3A).

**FIGURE 3:**
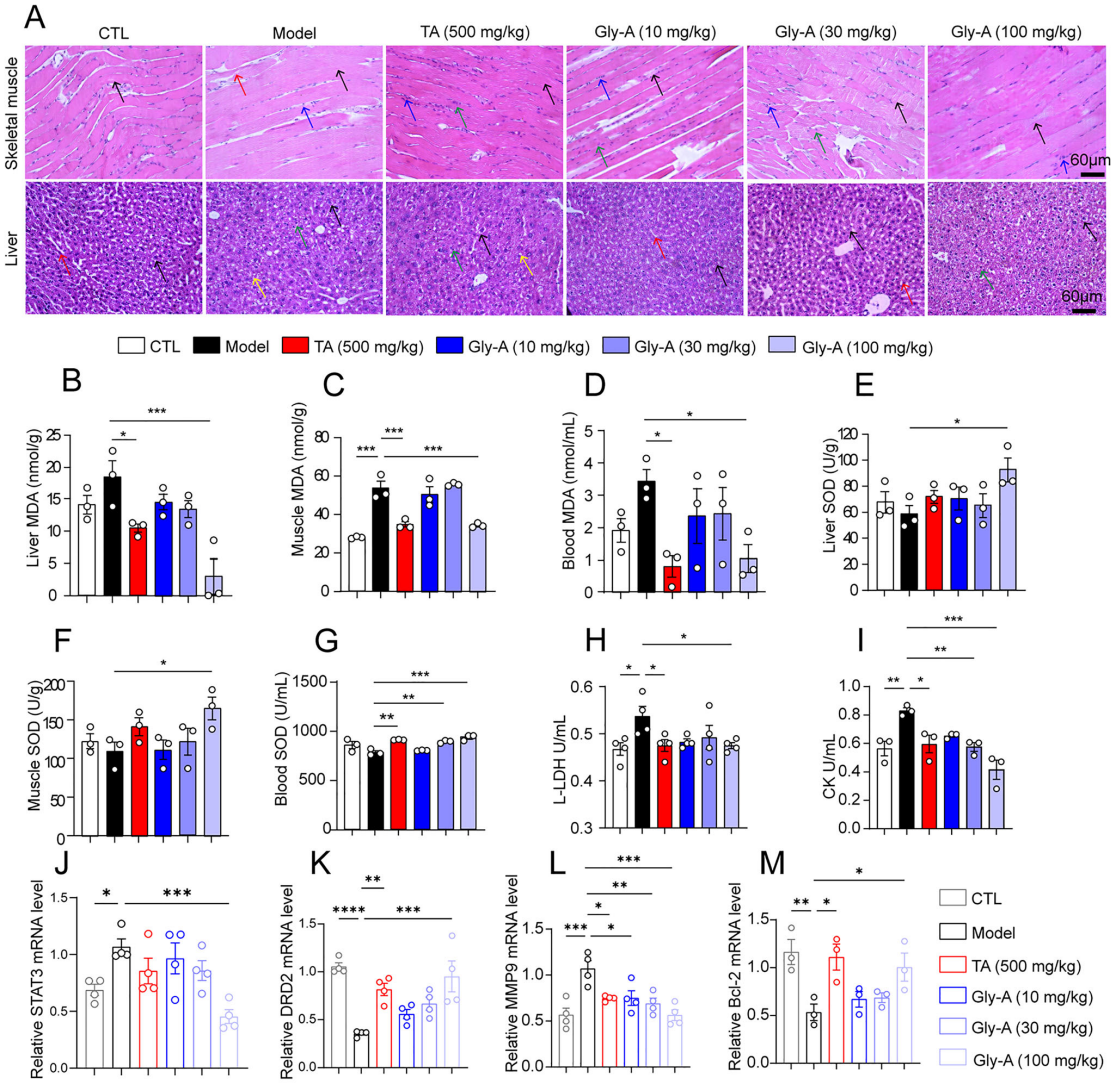
Gly‐A inhibited oxidative stress and enhanced skeletal muscle strength in vivo. (A) Photograph of HE staining of mouse skeletal muscle and liver tissue. (B–D) MDA activity of mouse muscle tissue, liver tissue, and mouse blood. (E–G) SOD activity of mouse muscle tissue, liver tissue, and mouse blood. (H and I) L‐LDH and CK activity of mouse blood. CK, creatine kinase; L‐LDH, l‐lactate dehydrogenase. (J–M) qRT‐PCR results of STAT3, DRD2, MMP9, and Bcl‐2 expression in hippocampus. U/g (tissue weight). CTL, control group; Gly‐A, glycyrrhizic acid; TA, taurine (*p < 0.05, **p < 0.01, ***p < 0.001).

SOD and MDA are important biochemical indicators related to fatigue (Y. Sun et al. 2022). According to the results of RNA sequencing, the response to stimulus and stress was closely associated with Gly‐A treatment for fatigue. We measured these two indicators (SOD and MDA) to evaluate the anti‐fatigue effects of Gly‐A. In the serum, liver, and muscle of the exercise‐fatigue mice, MDA levels (Figure 3B–D) were markedly decreased by Gly‐A and TA, while SOD levels significantly increased (Figure 3E–G). In the serum of the exercise‐fatigue mice, L‐LDH (L‐Lactate Dehydrogenase) and CK (Creatine Kinase) levels (Figure 3H,I) were markedly decreased by Gly‐A and TA (Figure 3H,I). qRT‐PCR was performed to quantify the mRNA expression levels of molecular docking targets including STAT3, DRD2, MMP9, Jun and BCL2 in the hippocampus (Figure 3J–M, Figure S1A). These data suggested that Gly‐A inhibits oxidative stress and enhances skeletal muscle strength in vivo.

### 4.4 Gly‐A Suppressed Inflammation by Targeting NF‐κB‐NLRP3 Signaling Pathway in Microglia

The network pharmacology analysis and RNA sequencing have identified that the inflammatory response pathway was an important target of Gly‐A in combating fatigue. Based on. KEGG enrichment analysis of RNA indicated that the NF‐κB‐NLRP3 signaling pathway is a crucial inflammatory response pathway. NF‐κB regulates the expression of NLRP3 and iNOS, which are involved in mitochondrial biogenesis, energy supply, and cell apoptosis (Zou et al. 2020). Both iNOS and TNF‐α modulate cell activity and inflammation (Im 2020), while IL‐1β, regulated by NF‐κB, enhances cell stress and the inflammatory response (B. Zhang et al. 2023). IF analysis showed that, following treatment with Gly‐A, the expression of NF‐κB decreased in the hippocampal CA1 (Figure 4A,B). Meanwhile, Gly‐A treatment significantly decreased the protein expression of NF‐κB, NLRP3, and iNOS in the hippocampus by Western blot (WB) analysis (Figure 4C–F). Additionally, qRT‐PCR was conducted to examine the mRNA expression of inflammatory factors such as IL‐1β in the hippocampus and cortex (Figure 4G–J, Figure S1B–F). The results indicated that the mRNA levels of IL‐1β, TNF‐α, and IL‐6 were significantly suppressed, while IL‐10 and IL‐4 were significantly increased after Gly‐A treatment (Figure 4G–J). Furthermore, WB analysis showed that the protein expression levels of NLRP3 and iNOS in the cortex of exercise‐fatigue mice were dose‐dependently decreased after Gly‐A treatment (Figure 4K–M). Both KEGG enrichment analysis and RNA sequencing also highlighted significant enrichment in the JAK‐STAT signaling pathway. To examine the association between Gly‐A and this signaling pathway, we conducted qRT‐PCR analyses to evaluate the cortical mRNA expression levels of JAK1 and STAT3. The results revealed that Gly‐A treatment markedly reduced the expression of both genes (Figure 4N,O). These results found that Gly‐A inhibits the inflammatory reaction in acute exercise‐fatigue mice.

**FIGURE 4:**
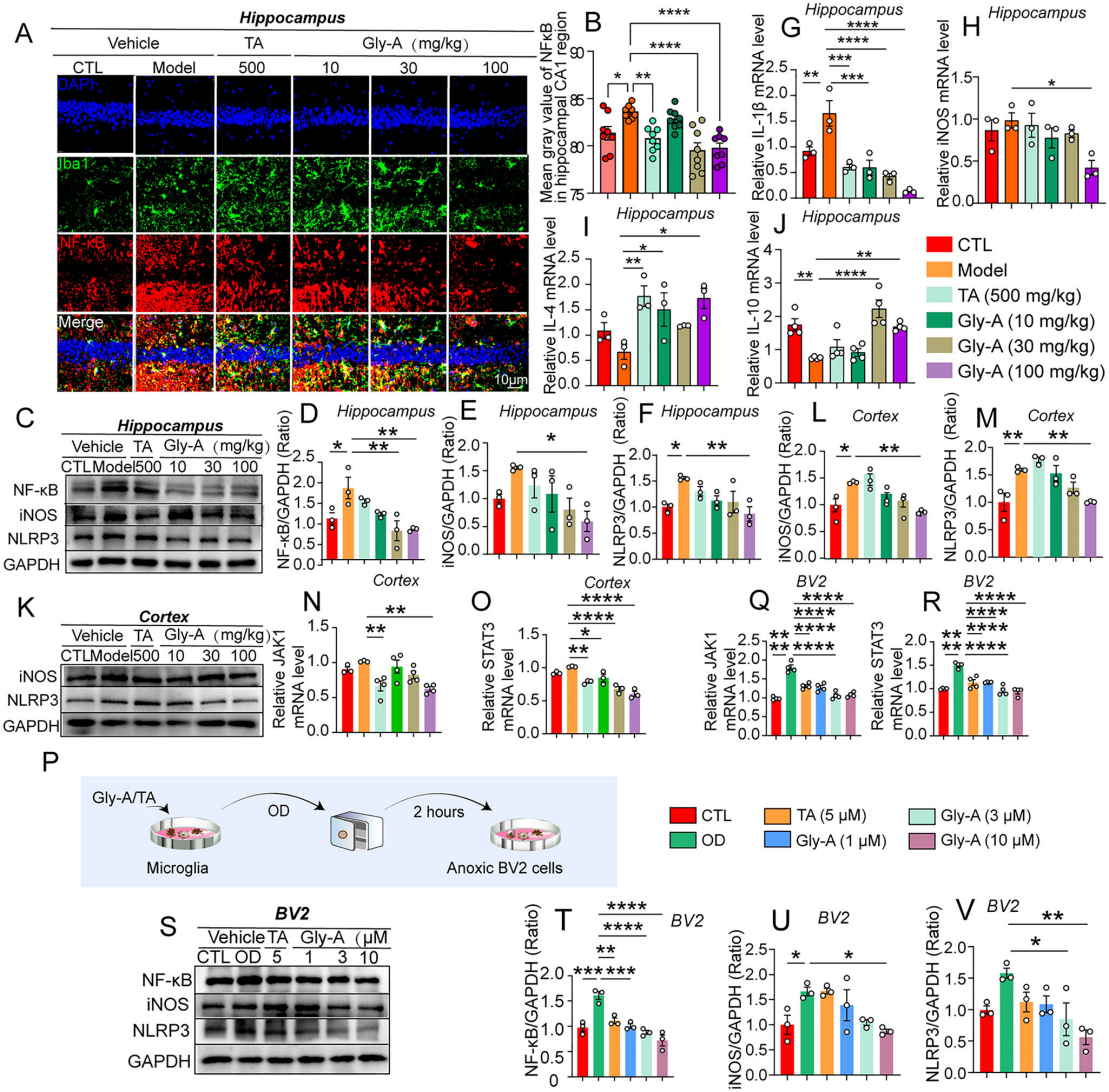
Gly‐A suppressed inflammation by targeting NF‐κB‐NLRP3 signaling pathway in microglia. (A and B) Immunofluorescence of NF‐κB and Iba1 in hippocampal CA1 region. (C–F) Western blot of NF‐κB, NLRP3, and iNOS in hippocampus. Data are normalized to β‐actin. (G–J) qRT‐PCR results of IL‐1β, iNOS, IL‐10, and IL‐4 expression in hippocampus. (K–M) Western blot of NLRP3 and iNOS in cortex. Data are normalized to β‐actin. (N–O) qRT‐PCR results of JAK1 and STAT3 expression in cortex. (P) Flowchart of Gly‐A treat OD model of BV2 cell. (Q and R) qRT‐PCR results of JAK1 and STAT3 expression in in BV2 cell with Gly‐A after OD for 2 h. (S) Western blot of NF‐κB, NLRP3 and iNOS in BV2 cell with Gly‐A after OD for 2 h. (T–V) Quantitation of NF‐κB, NLRP3, and iNOS. Data are normalized to GAPDH (*p < 0.05, **p < 0.01, ***p < 0.001, ****p < 0.0001).

Within neurological disorders, microglia of different phenotypes undertake different functions. Specifically, while the M1 phenotype is associated with harmful effects, the M2 phenotype confers neuroprotection (Xia et al. 2015). To assess the anti‐fatigue mechanism of Gly‐A on microglia phenotypes, we used an OD cell model to simulate the fatigue model in vitro (Figure 4P). First, a CCK‐8 test was conducted to measure the cytotoxic effects and determine the optimal duration of OD treatment for microglia in the Gly‐A group (Figure S1G–N). Our analysis indicated a marked elevation in JAK1 and STAT3 mRNA levels in the OD group relative to the non‐stimulated control, whereas the Gly‐A (10 µM) group exhibited a notable decline in expression (Figure 4Q,R). Additionally, WB analysis showed that OD stimulation led to the upregulation of proteins in the NF‐κB‐NLRP3 signaling pathway, including NF‐κB, NLRP3, and iNOS in the OD group, whereas these proteins were significantly downregulated in the Gly‐A (10 µM) group (Figure 4S–V). The qRT‐PCR results showed that Gly‐A inhibited the M1 microglia phenotype while significantly enhanced the M2 microglia phenotype (Figure S1a–f). Therefore, these data indicated that Gly‐A suppresses inflammation by regulating microglia phenotypes through the NF‐κB‐NLRP3 signaling pathway.

### 4.5 Gly‐A Effectively Inhibited Apoptosis by Targeting the Apoptotic Signaling Pathway in Neuron

Our toluidine blue staining results showed that Gly‐A increased the level of chromophilic substance in the hippocampus of exercise‐fatigue mice (Figure 5A,B). Additionally, qRT‐PCR analysis revealed a significant increase in Bcl‐2 mRNA expression, along with a decrease in Bax expression in response to Gly‐A treatment (Figure 5C,D). These results suggested that Gly‐A might exhibit anti‐fatigue activity in vivo by inhibiting the apoptotic signaling pathway.

**FIGURE 5:**
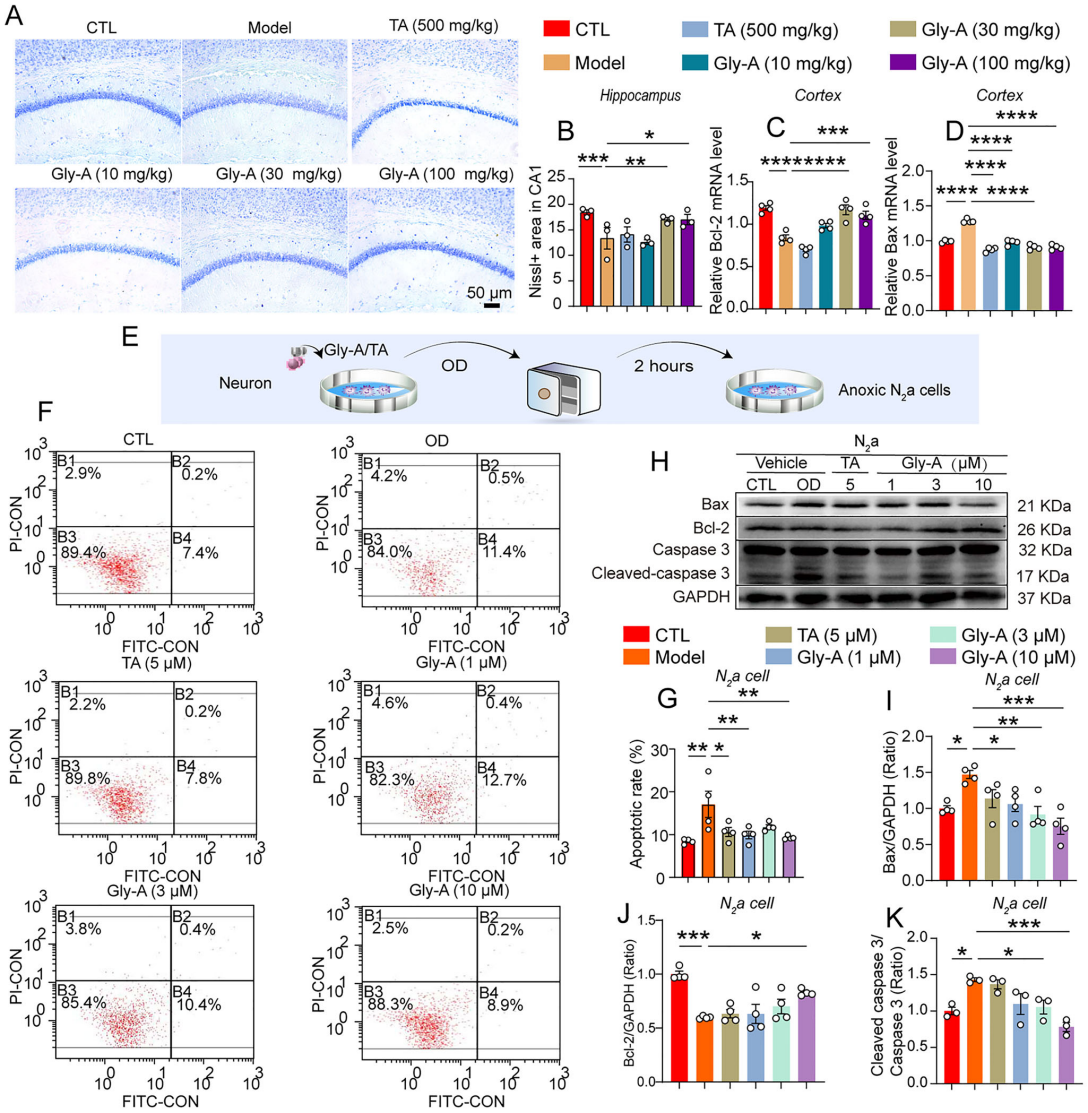
Gly‐A effectively inhibited apoptosis by targeting mitochondria apoptotic signaling pathway in neuron. (A) Photograph of toluidine blue staining in hippocampal CA1 region. (B) Quantitative analysis of toluidine blue staining in hippocampal CA1 region. (C and D) qRT‐PCR results of Bcl‐2 and Bax expression in cortex. (E) Flowchart of Gly‐A treat OD model of N_2_a cell. (F–G) Flow cytometry and quantitation of Gly‐A treat OD model of N_2_a cell. (H–K) Western blot and quantitation of Bax, Bcl‐2, and Caspase 3 in N_2_a cell with Gly‐A after OD for 2 h. Data are normalized to GAPDH. CTL, control group; Gly‐A, glycyrrhizic acid; OD, oxygen deficit group; TA, taurine (*p < 0.05, **p < 0.01, ***p < 0.001, ****p < 0.0001).

To further investigate the anti‐fatigue mechanism of Gly‐A in neuronal cells, we utilized N_2_a cells and established an OD model based on our cytotoxicity assessments in microglia (Figure 5E). The results of flow cytometry showed that Gly‐A (10 µM) significantly inhibited apoptosis in neuronal cells following 2 h of OD (Figure 5F,G). Subsequently, WB analysis was used to measure the expression levels of Bcl‐2, Bax, and Caspase 3. The data demonstrated that Gly‐A treatment reduced the expression of Bax and Caspase 3 proteins while increasing Bcl‐2 expression compared to the control group (Figure 5H–K). Therefore, our results suggested that Gly‐A inhibits neuronal cell apoptosis through suppressing apoptotic signaling pathway

### 4.6 Gly‐A Significantly Inhibited CF in the Mice Model

After confirming Gly‑A's significant anti‑fatigue effects in the acute exercise‑fatigue model, we evaluated its efficacy in CF. A CF mouse model was established and treated with Gly‑A via oral gavage or intraperitoneal injection (Figure 6A). Nandro was demonstrated to attenuate CF in animal models subjected to overload stress (Ben‐Zeev et al. 2025), and thus served as a positive control for assessing therapeutic efficacy in our study.

**FIGURE 6:**
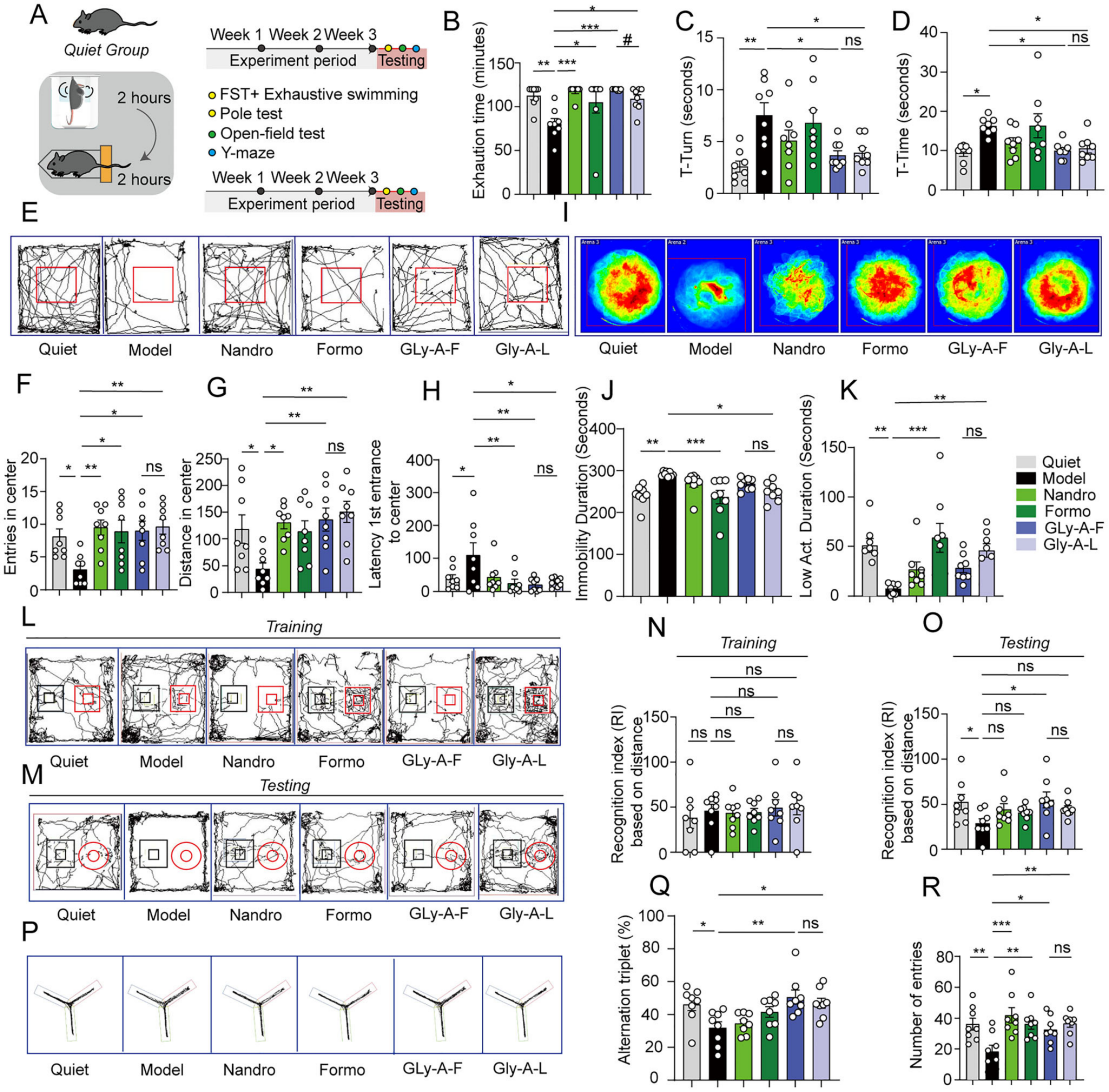
Gly‐A significantly inhibited CFS in the mice model. (A) Establishment of CFS animal model. (B) Exhausting time in FST in the 21st day. (C) Duration of T‐Turn in pole test. (D) Duration of T‐Time in pole test. (E) Trajectory diagram of OFT in CFS mice. (F) Entries in center of OFT in CFS mice. (G) Distances in center of OFT in CFS mice. (H) Latency the 1st entrance to center of OFT in CFS mice. (I) Trajectory diagram of FST in CFS mice. (J) Immobility duration of FST in CFS mice. (K) Duration of low action of FST in CFS mice. (L) Trajectory diagram of object recognition test in training period. (M) Trajectory diagram of object recognition test in testing period. (N) Recognition index based on distance in training period. (O) Recognition index based on distance in testing period. (P) Trajectory diagram of Y maze experiment in CFS mice. (Q) Y maze behavioral experiment. Spontaneous alternation triplet (%). (R) Y maze behavioral experiment. Number of arms entered. CFS: chronic fatigue syndrome; Formo, formoterol; Gly‐A, glycyrrhizic acid; Gly‐A‐F: Gly‐A for oral use; Gly‐A‐I: Gly‐A for injected use; Nandro, nandrolone; TA, taurine; T‐Time: duration taken to reach the bottom from the top; T‐Turn: the latency of the mice to auto‐turn (*p < 0.05, **p < 0.01, ***p < 0.001, ****p < 0.0001) (^ns^ *p*>0.05,^#^ *p* < 0.05; Gly‐A‐F vs. Gly‐A‐L).

Body weight remained unchanged across all treatment groups (Figure S2). In the CF model, oral and intraperitoneal administration of Gly‑A produced comparable improvements in neurological function. Compared with healthy controls, model mice exhibited a significantly shorter exhaustion swimming time, confirming successful induction of physical fatigue. All Gly‑A–treated groups demonstrated marked increases in swim endurance relative to the untreated CF mice (Figure 6B). In the pole test, both oral and injected Gly‑A significantly prolonged T‑Time and T‑Turn versus model mice (Figure 6C,D). CFS is characterized by CF lasting more than six months, accompanied by physical exhaustion, cognitive impairment, and diminished occupational or functional capacity. To assess mental fatigue, we used the OFT and FST. Model mice displayed reduced activity in both assays compared to quiescent controls (Figure 6E–K). The Gly‐A‐orally mice showed significant improvements in OFT performance (Figure 6E–H), whereas FST immobility remained unchanged (Figure 6I–K). Notably, injection of Gly‑A or Formo restored both OFT and FST behaviors (Figure 6E–K). Given that neurocognitive impairment is a hallmark of CFS, we evaluated cognition using the novel object recognition and Y‑maze tests. Oral Gly‑A significantly enhanced object recognition memory (Figure 6L–O) and improved Y‑maze spatial alternation compared to model mice (Figure 6P–R). These findings demonstrated that Gly‑A, via both oral and injected routes, exerts significant anti‑CFS effects in mice.

### 4.7 Gly‐A Exhibits Anti‐CF Effect by Targeting NF‐κB‐NLRP3 Signaling Pathway

Building on our acute exercise–fatigue findings, we next measured SOD and MDA as oxidative‐stress markers to assess Gly‑A's anti‑CF activity. In serum, liver, and muscle of CF mice, Gly‑A treatment significantly lowered MDA and CK levels and elevated SOD activity (Figure 7A–G). We then used qRT‑PCR to quantify hippocampal mRNA expression of inflammatory mediators: Gly‑A markedly downregulated IL‑1β, iNOS, and IL‑6 transcripts while upregulating anti‑inflammatory IL‑10 and IL‑4 (Figure 7H–L). Oral Gly‑A also significantly reduced NLRP3 and NF‑κB mRNA in the hippocampus compared to untreated CF mice (Figure 7M,N), and IF confirmed decreased NLRP3 protein in the CA1 region (Figure 4R,S). Finally, qRT‑PCR analysis of apoptosis genes showed that Gly‑A lowered pro‑apoptotic Bax and Bad expression and increased anti‑apoptotic Bcl‑2 levels (Figure 7O,Q). Together, these results demonstrated that oral Gly‑A inhibits the NF‑κB/NLRP3 axis in CF mice.

**FIGURE 7:**
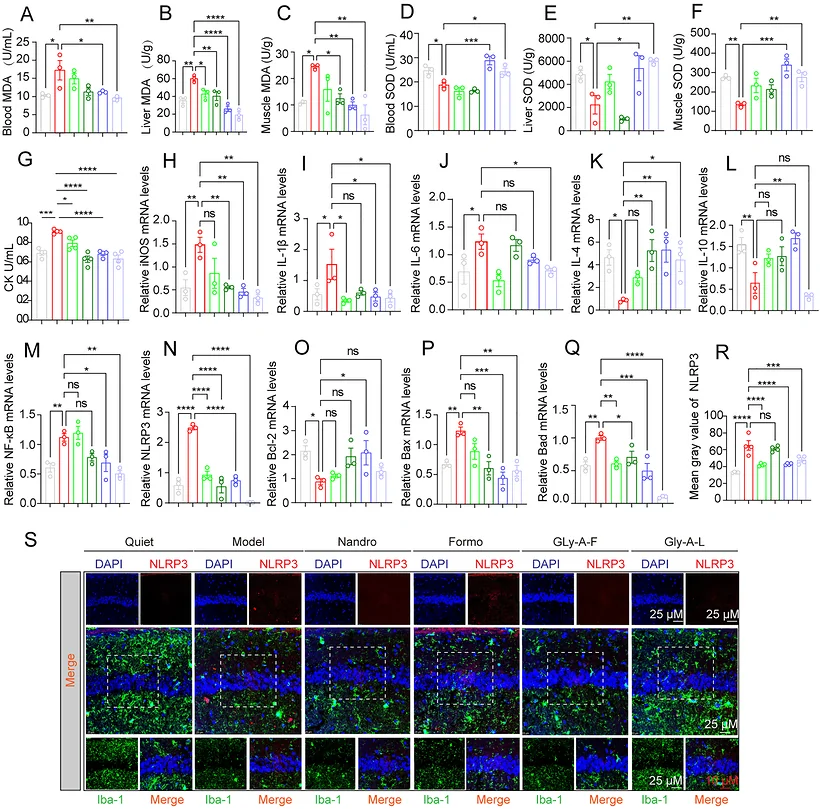
Gly‐A exhibits anti‐CFS effect by targeting NF‐κB‐NLRP3 signaling pathway to inhibit mitochondrial apoptosis signaling pathway. (A–C) MDA activity of mouse muscle tissue, liver tissue, and mouse blood. (D–F) SOD activity of mouse muscle tissue, liver tissue, and mouse blood. (G) CK activity of mouse blood. (H–L) Quantitation of iNOS, IL‐1β, IL‐6, IL‐10, and IL‐4 mRNA expression in hippocampus. (M and N) Quantitation of NF‐κB, NLRP3 mRNA expression in hippocampus. (O–Q) Quantitation of Bax, Bad, and Bcl‐2 mRNA expression in hippocampus. (R and S) IF of NLRP3 and Iba1 in hippocampal CA1 region. CFS: chronic fatigue syndrome (*p < 0.05, **p < 0.01, ***p < 0.001, ****p < 0.0001).

## 5 Discussion

Gly‐A, a time‐honored medicinal plant from Chinese tradition and a member of the legume family, is celebrated for its efficacy in dissipating heat and detoxifying the body. Previous studies have demonstrated that Gly‑A alleviates oxidative stress and exerts neuroprotective effects, showing efficacy in models of chronic liver disease (M. Guo et al. 2023), early gastrointestinal disease (X. Wang, Shi, et al. 2025), Parkinson's disease (Ojha et al. 2016), and other neurological conditions. However, its potential to treat acute and chronic exercise‑fatigue remains unclear. In this study, we evaluated Gly‑A's therapeutic effects on both acute and chronic exercise‑fatigue in mice and determined the optimal dosage and route of administration. Our results showed that Gly‑A significantly enhanced motor performance and exercise endurance, and alleviated depressive behaviors and neurocognitive deficits. These findings suggest that Gly‑A not only combats acute exercise‑fatigue but also provides neuroprotective benefits in CFS.

In behavioral tests, Gly‑A significantly reversed acute exercise‑fatigue only when administered by intraperitoneal injection. In the CF model, however, both intraperitoneal and oral Gly‑A improved motor performance, reduced anxiety and depressive‐like behaviors, and preserved neurocognitive function. This discrepancy likely reflects fundamental differences between the two models: the acute fatigue paradigm induces only short‐term physical exhaustion, whereas the CF model replicates both physical and mental fatigue—including pain, cognitive impairment, and mood disturbances (Thapaliya et al. 2025)—over an extended period. In our preliminary studies, a single intraperitoneal dose of 100 mg/kg Gly‑A markedly alleviated exercise‑induced fatigue, and oral administration produced equivalent therapeutic effects in CF mice.

Recent advances in network pharmacology and RNA sequencing have enabled the identification of core therapeutic targets and the elucidation of drug mechanisms. To investigate how Gly‑A combats fatigue, we focused on its effects on inflammation and oxidative stress in a mouse model of acute and chronic exercise‑fatigue. Acute strenuous exercise accelerates the production of ROS, triggering oxidative stress and inflammation. Numerous in vivo studies have shown that CF patients exhibit elevated ROS levels. Cellular responses such as proliferation, differentiation, and apoptosis are regulated by the ROS generated by the cells. A study has revealed that HBV infection enhances ROS production and causes oxidative stress in host cells (Ning et al. 2023). MDA and SOD are ideal indicators to evaluate and to express oxidation status and the production of ROS (J. Guo et al. 2021). Thus, Accordingly, we measured MDA and SOD in the serum, muscle, and liver of CF mice. Gly‑A treatment significantly lowered MDA levels and increased SOD activity in all three tissues, consistent with previous findings. These results demonstrate that Gly‑A alleviates both acute and chronic exercise‑fatigue in mice by restoring redox balance.

Positive control drugs such as Nandro have been shown to alleviate CF in animal models subjected to overload stress (Ben‐Zeev et al. 2025). As reported, androgen treatment reduces mitochondrial ROS generation during muscle contraction, thereby decreasing tissue damage and TNF‑α production in both the plantaris and soleus muscles. In addition, Formo has been reported to improve the functional and structural properties of the soleus (Harcourt et al. 2007). Although Formo demonstrated a pronounced therapeutic effect on soleus muscle function, its efficacy in alleviating CF remains to be established. This report, together with our results, also verifies the function of in CF. In our study, we compared their effects with those of orally administered Gly‑A. Remarkably, Gly‑A not only relieved anxiety and depressive‐like behaviors in CF mice but also enhanced neurocognitive function. In contrast, Nandro yielded an obvious therapeutic effect on CF; however, it did not produce a statistically significant improvement in neurocognitive function. Formo reduced depressive behaviors but had minimal impact on cognitive deficits. Overall, Gly‑A consistently improved motor activity and neurocognitive performance in CF mice, offering a promising alternative to androgens and bronchodilators, which are banned by sports authorities due to their adverse reaction.

The NLRP3 inflammasome is widely recognized for its inflammatory role in triggering cell death. (Aranda‐Rivera et al. 2022). The oxidative burst of inflammatory cells during inflammation, in conjunction with mitochondrial dysfunction, exacerbates the production and accumulation of ROS, thereby promoting oxidative stress and neuronal damage, which may contribute to the development of neurological disorders (Kelley et al. 2019; Kim et al. 2019; Mohtadi et al. 2024; Qiu et al. 2023). In our study, we have demonstrated that despite the absence of a clear dose‐dependent response, Gly‐A is capable of inhibiting the activation of the NF‐κB/NLRP3 inflammasome axis. Our molecular docking and RNA sequencing results show that the Bcl‐2‐mediated apoptotic pathway is involved in Gly‐A's anti‐fatigue mechanism. These findings are consistent with our results, suggesting that Gly‐A can protect against brain inflammation and apoptosis from nerve cell damage following acute exercise‐fatigue. Therefore, our results suggest that Gly‐A may exert its anti‐fatigue effect by regulating the NF‐κB/NLRP3 signaling pathway and the apoptotic pathway.

The hippocampus plays a pivotal role in learning, memory, and pain modulation, and is particularly vulnerable to neuroinflammation, oxidative stress, and neurotransmitter dysregulation (Baktır 2025). The NF‐κB/NLRP3 inflammasome axis, identified through network pharmacology and RNA sequencing as a key regulator in alleviating acute exercise‐fatigue, was investigated for its neuroprotective potential in CF in our study. Mice administered with Gly‐A exhibited a reduced peripheral ROS response and increased levels of antioxidant enzymes. Here, our results have found that Gly‐A alleviates CF in mice, as shown by improved locomotor activity, delayed swimming exhaustion, and spontaneous alternation in the Y‐maze. These findings align with prior studies showing that elevated pro‐inflammatory cytokines and downregulation of anti‐apoptotic proteins contribute to neuronal damage in memory‐associated brain regions, including the hippocampus. This study shows that four weeks of forced swimming elevates pro‐inflammatory cytokine levels in the hippocampus of mice with CF. This dysregulated inflammatory response promotes activation of the NF‐κB/NLRP3 inflammasome signaling axis, thereby accelerating neuronal apoptosis and contributing to selective neuronal injury. Multiple independent studies consistently report elevated IL‐1β and TNF‐α levels in the brains of CFS mouse models (K. Wang, Shen, et al. 2025). This molecular dysregulation drives mitochondrial dysfunction, thereby promoting neuroinflammation, reactive gliosis, neuronal injury, nociceptive hypersensitivity, and hippocampal‐dependent memory impairment (Sun et al. 2025). Consistent with these findings, Gly‐A treatment significantly reduced hippocampal levels of IL‐1β and TNF‐α in CF mice. Moreover, a substantial body of experimental and clinical evidence supports a causal involvement of neuroinflammatory and apoptotic pathway dysregulation in the central nervous system in both the initiation and progressive intensification of fatigue symptoms (Khodir et al. 2025). In this study, Gly‐A treatment significantly downregulated the expression of pro‐apoptotic proteins in the brain, providing mechanistic evidence that neuroinflammation drives neuronal apoptosis and that sustained inflammatory activation exacerbates cumulative neural damage. Our study has identified optimal administration regimens of Gly‐A for both acute and chronic exercise‐fatigue. This finding provides robust evidence to support Gly‐A is regarded as a promising candidate for development into an innovative and potent anti‐fatigue agent.

## 6 Limitations

This study has several limitations. First, mouse models were established to simulate acute and chronic exercise‐fatigue. These animal models were experimentally induced to recapitulate key behavioral and physiological symptoms associated with acute and chronic exercise‐fatigue. However, the symptoms produced through these interventions may not fully reflect the underlying pathophysiological mechanisms observed in human patients. Moreover, when establishing mouse models of fatigue, it is essential to closely monitor the animals' physiological status. During the experimental procedure, mice in the acute exercise‐fatigue model are susceptible to death due to exhaustion from intense physical exertion, whereas those in the CF model are at risk of mortality from drowning or asphyxiation during behavioral challenges. In addition, our study only focused on evaluating neurobehavioral performance and a panel of molecular biomarkers associated with fatigue. Nevertheless, the plasma concentration of Gly‐A in mice was not determined, constituting a limitation that precludes direct correlation between observed effects and systemic drug exposure. This critical limitation will be addressed in future investigations through comprehensive pharmacokinetic profiling, enabling the establishment of dose–response relationships. Finally, previous studies have shown that peripheral and immune cell infiltration are closely associated with tumorigenesis and cardiovascular disease onset (L. Li et al. 2024; Zhu et al. 2025). However, the specific causal relationship between the microglia‐mediated NF‐κB/NLRP3 inflammasome axis and acute and chronic exercise‐fatigue remains to be fully elucidated, necessitating further investigation in future studies.

## 7 Conclusions

In summary, our study demonstrated that Gly‑A exerts significant anti‑fatigue effects in mouse models of acute and chronic exercise‑fatigue by modulating the NF‑κB/NLRP3 signaling axis and the apoptotic pathway. We identified that inhibition of the NF‐κB/NLRP3 inflammasome axis contributes to the neuroprotective effects of Gly‐A in neuroinflammatory diseases. Gly‑A alleviates peripheral fatigue by restoring peripheral redox homeostasis and metabolic function. It also alleviates central fatigue by suppressing neuroinflammation in the hippocampus and cerebral cortex. These findings suggest that Gly‑A could serve as a promising dietary supplement for managing neuroinflammatory diseases including both acute and chronic exercise‑fatigue. Our study provides valuable insights and practical recommendations to inform future double‐blind, multicenter, randomized controlled trials of Gly‐A.

## Author Contributions

**Xin‐ying Cai**: study design, manuscript writing, methodology, analysis. **Lu Peng**: validation. **Jian‐bo Jia**: funding acquisition. **Hong‐tao Liu**: methodology. **Li‐chao Yang**: writing – review and editing. **Yan‐chao Jiao**: software, supervision. **Wen‐jun Li**: software. **Xiao‐ning Lin**: methodology. **Cai‐xia Chen**: software. **Ying Li**: writing – review and editing, project administration.

## Funding

This study was supported by grants from the Major Scientific Research Program for Young and Middle‐aged Health Professionals of Fujian Province, China (No.: 2023ZQNZD019), Fujian Province Science and Technology Innovation Joint Fund Project (Nos: 2024Y9722, 2024Y9700), Guiding Medical and Health Projects of Xiamen (3502Z20244ZD1144), The Fujian Province Science and Technology Plant Project (2023D023), Natural Science Foundation of Hebei Province (H2024205002).

## Ethics Statement

All animal procedures received approval from the Medical Ethics Committee of Xiamen Medical College (20231110001) and were conducted under the supervision of its affiliated laboratory.

## Conflicts of Interest

The authors declare no conflicts of interest.

## Supporting information




**Supplementary Material**: brb371773‐sup‐0001‐FigureS1‐S2.docx

**Supplementary Material**: brb371773‐sup‐0002‐TableS1‐S2.docx

## Data Availability

All data or any additional information reported in this paper is available from the lead contact upon request.
