# Supplementary material for: Glycyrrhizic Acid Relieves Fatigue Induced by Acute and Chronic Exercise in Mice via Inhibition of the NF‐κB/NLRP3 Signaling Pathway
Source: Brain Behav. 2026 Sep 27;16(10):e71773. doi: 10.1002/brb3.71773 (PMC13617204; doi:10.1002/brb3.71773)
Supplement: Supplementary file 1 [file BRB3-16-e71773-s001.docx]

Supplementary Table 1 Chemical and reagent in this study

| Glycyrrhizic acid | A0037 | Chengdu must bio-technology CO., LTD, China |
| --- | --- | --- |
| Nandrolone decanoate | MFCD00135100 | Shanghai Macklin Technology Co., LTD, China |
| Taurine | MFCD00008197 | Shanghai Macklin Technology Co., LTD, China |
| Formoterol fumarate | F129812 | Shanghai Aladdin Biochemical Technology Co., LTD, China |
| SOD assay kits | [BC5165](https://www.solarbio.com/goodsInfo?id=89337) | Beijing Solaibao Technology Co., LTD, China |
| MDA assay kits | [BC6415](https://www.solarbio.com/goodsInfo?id=315862) | Beijing Solaibao Technology Co., LTD, China |
| BCA protein assay kit | [PC0020](https://www.solarbio.com/goodsInfo?id=64504) | Beijing Solaibao Technology Co., LTD, China |
| CK assay kits | BC1145 | Beijing Solaibao Technology Co., LTD, China |
| L-LDH assay kits | BC0685 | Beijing Solaibao Technology Co., LTD, China |
| SDS-PAGE gel kit | 20328ES | Yeasen, China |
| iNOS antibody | 22226-1-AP | Proteintech, Wuhan, China |
| NLRP3 antibody | 30109-1-AP | Proteintech, Wuhan, China |
| NF-κB antibody | 10745-1-AP | Proteintech, Wuhan, China |
| Bcl-2 antibody | 60178-1-Ig | Proteintech, Wuhan, China |
| Caspase 3 antibody | 19677-1-AP | Proteintech, Wuhan, China |
| Bax antibody | 50599-2-Ig | Proteintech, Wuhan, China |
| GAPDH antibody | 60004-1-Ig | Proteintech, Wuhan, China |
| Iba-1 antibody | 019-19741 | FUJIFILM Wako Chemical corporation, OSAKA, Japan |
| DMEM (high glucose) medium | C11995500bt | Gibco, USA |
| bovine serum (FBS) | 10099141C | Gibco, USA |
| HE Stain Kit | C0105S | Beyotime, China |
| Goat anti-rabbit | RGAR002 | Proteintech ,Wuhan, China |
| Goat anti-mouse | RGAM004 | Proteintech ,Wuhan, China |
| CCK-8 assay | K1018 | Apexbio, China |
| RIPA Buffer | R0010 | Beijing Solaibao Technology Co., LTD, China |
| SDS-PAGE Loading Buffer | P1040 | Beijing Solaibao Technology Co., LTD, China |
| SYBR Green I dye | 11203ES08 | Yeasen, China |
| Annexin V-FITC/PI Apoptosis Detection Kit Annexin V-FITC/PI | 40302ES60 | Yeasen, China |
| DAPI Solution | C0065 | Beijing Solaibao Technology Co., LTD, China |
| Three-standard four-color multiplex fluorescence staining kit | AFIHC024 | AiFang Biological, China |
| BV2 cell | TCM-C718 | Suzhou Starfish Biotechnology Co., Ltd. |
| Neuro-2a (N2a cell) | TCM-C750-UD | Suzhou Starfish Biotechnology Co., Ltd. |

Supplementary Table 2 Primer used for real-time PCR in this study

| Genes | Forward primer(5'→3') | Reverse primer(5'→3') |
| --- | --- | --- |
| STAT3 | AATCTCAACTTCAGACCCGCCAAC | GCTCCACGATCCTCTCCTCCAG |
| JAK1 | CCCACTCCTTGATGCCAGTTCAC | CATGTCCGTCTTGCTCCGTCTTG |
| GAPDH | AGGTCGGTGTGAACGGATTTG | GGGGTCGTTGATGGCAACA |
| IL-1β | CACTACAGGCTCCGAGATGAACAAC | TGTCGTTGCTTGGTTCTCCTTGTAC |
| IL-4 | TACCAGGAGCCATATCCACGGATG | TGTGGTGTTCTTCGTTGCTGTGAG |
| IL-6 | CTCCCAACAGACCTGTCTATAC | CCATTGCACAACTCTTTTCTCA |
| IL-10 | TTCTTTCAAACAAAGGACCAGC | GCAACCCAAGTAACCCTTAAAG |
| iNOS | ATCTTGGAGCGAGTTGTGGATTGTC | TAGGTGAGGGCTTGGCTGAGTG |
| TNF-α | ACCCTCTGCTCTACGAATCACTCTG | CGCCATCCACCACAGCATACAG |
| BCl-2 | CTTCTCTCGTCGCTACCGTC | CAATCCTCCCCCAGTTCACC |
| Bad | CCGTCCCTGCTGATGAATGT | AGAGTTTGAGCCGAGTGAGC |
| Bax | CACCTGAGCTGACCTTGGAG | CAAACATGTCAGCTGCCACC |
| NLRP3 | AAACCCACCAGTGTGCAAGA | CAAAGGCCCCTTGTAGCTCA |
| NF-κB | CTGTCACAGACGCTGTCACT | AAGTGATCCAGGCAGCCTTC |
| JUN | CTTCTACGACGATGCCCTCAACG | GCCAGGTTCAAGGTCATGCTCTG |
| MMP9 | CGCCACCACAGCCAACTATGAC | CTGCTTGCCCAGGAAGACGAAG |
| DRD2 | CAGTGAACAGGCGGAGAATGGATG | GGTGGTGACTGGGAGGGATGG |
