# Supplementary material for: Glycyrrhizic Acid Relieves Fatigue Induced by Acute and Chronic Exercise in Mice via Inhibition of the NF‐κB/NLRP3 Signaling Pathway
Source: Brain Behav. 2026 Sep 27;16(10):e71773. doi: 10.1002/brb3.71773 (PMC13617204; doi:10.1002/brb3.71773)
Supplement: Supplementary file 2 [file BRB3-16-e71773-s002.docx]

**Supplementary material**

**Supplementary figure 1:** (A) qRT-PCR results of Jun expression in hippocampus. (B-F) qRT-PCR results of IL-1β, TNF-α, IL-6, IL-10 and IL-4 expression in cortex. (G-I) Cell viability assay (CCK-8) for BV2 cell with Gly-A with different concentrations for 12, 24 and 48 hours. (J-N) Cell viability assay (CCK-8) for BV2 cell with Gly-A and TA with different concentrations after OD for 1, 2 and 4 hours. (a-f) qRT-PCR results of iNOS, IL-6, IL-1β, TNF-α, IL-10 and IL-4 expression. CTL, control group; Gly-A, Glycyrrhizic acid; TA, taurine; OD, oxygen deficit group. CTL, control group; Gly-A, Glycyrrhizic acid; TA, taurine; OD, oxygen deficit group; (* p < 0.05, ** p < 0.01, *** p < 0.001, **** p < 0.0001).

**Supplementary figure 2:** (A) Changes in body weight of CFS mice. Nandro, Nandrolone; Formo, Formoterol; Gly-A-F: Gly-A for oral use; Gly-A-I: Gly-A for injected use; CFS: chronic fatigue syndrome.
